# The Cure of Squinting by the Use of Prismatic Spectacles

**Published:** 1853-11

**Authors:** T. Spencer Wells


From the London Lancet.

*The cure of Squinting by the use of Prismatic Spectacles.* By T. Spencer Wells, F.R.C.S.

Dr. Kurke, a Dutch physician, first recommended prismatic spectacles for the cure of squinting. He has recorded one case cured by their use in the Dutch journals. Dr. Von Grafe, of Berlin, has since employed them very extensively. During a recent visit to Berlin, I had frequent opportunities of observing their effects upon his patients, and I think that the result of his experience should be made known to the profession in England.

The glasses are fitted in ordinary spectacle frames. They are simple prisms of various degrees from 1 to 20. It would be possible to make them achromatic; but I have only seen the ordinary ones in use.

The operation upon the sound eye, as explained by Dr. Von Grafe, is as follows:—When a prismatic glass is held before one eye on any point of sight in the converging direction of the optic axis, the light falling upon this eye, is diverted from its former course, and no longer arrives upon the macula lutea, but forms a more or less excentric picture, according to the refracting power of the prism. From its position, this is no longer combined with the central picture on the retina into one perception, but is perceived separately. Thus the object upon which the optic axes converge is seen double.

Theoretically this phenomenon should be observed when a prism of very moderate power is used; but observation teaches us, on the contrary, that no diplopia follows, when weak prisms are employed, especially if the base be directed outwards. This might be explained in two ways. Either the picture on one retina is suppressed, or the eye which sees through the prism takes a new position, which is not perceived by the observer, so that the picture is not formed excentrically, but falls, like that of the other eye, upon the macula lutea. The improbability of the first supposition at once appears from the fact that no diplopia is produced by weak prisms, while more powerful ones produce it at once, and the greater the excentric position of the picture the more easily it would be suppressed. The truth of the second explanation is established by a more exact observation of the position of the eyes. On applying the prism we see the optic axes deviate from their former position, and return to it as the prism is removed. At the moment of removal the object is seen double, because both axes are not directed upon it. Thus in order to prevent diplopia, an involuntary strabismus occurs, and we cau produce this in any direction by corresponding positions of the prism, but most decidedly so inwards, less so outwards, much less so downwards, and least of all upwards. We can also produce strabismus in this manner in diagonal directions.

It follows that by the use of prismatic glasses, we have the power of altering the tension of any given muscle of one eye without producing any alteration in the other. This is the peculiar advantage which none of the ordinary orthopœdic means formerly employed possessed. On the contrary, the result hoped for from their employment was not only frequently frustrated by the movements of association of the two eyes, but sometimes, as in cases of recent muscular paralysis. an effect directly the reverse of that desired was brought about.

The increased contraction called for from the relaxed muscle by the use of prismatic glasses is the source of their curative power. For example, in a case of convergent strabismus with diplopia a prism with its base directed outwards alters the position of the excentric picture on the retina of the squinting eye so greatly, and brings it so near the macula lutea, that single vision follows any voluntary power conveyed to the abductor muscle. Consequently, the angle of the squint is somewhat diminished. As it becomes less. and the power of the abductor increases, prisms must be used gradually diminishing in power, until at last a perfectly accurate corresponding position of the eye is attained at all distances,—in other words, the squint is perfectly cured. I have seen patients of

Dr. Von Grafe's who were thus completely cured in about six weeks, commencing with strong glasses of the numbers from 15 to 20, and gradually wearing them less and less powerful. They are principally applicable in young persons, who squint but slightly; and in cases of diplopia binocularis, where the abnormal position of one eye is only observed when an object some feet distant is regarded, they are the only certain means of cure.

In more marked degrees of strabismus the muscle must be divided, because the use of strong prisms, and the efforts of the patient to avoid diplopia, become very troublesome; and if the union of the two images causes too great an effort, an effect is produced exactly the opposite of that desired; for if the diplopia cannot be removed, the double images separate still further from each other, because when distant, they are not so intolerable as when near.

In many cases after operations for the cure of strabismus by division of the muscle in one or both eyes, although great improvement follows, the cure is not perfect. Some degree of squint still persists in one eye, and probably some diplopia when objects at certain distances from the eye are attentively regarded. In such cases, the prismatic glasses suffice to complete the cure commenced by the operation. I saw several instances in which this proved to be the case in the practice of Dr. Von Grafe.

I have patients under my care at present who are wearing these spectacles, and I shall take a future opportunity of making the results known. Messrs. Watkins and Hill, opticians, of Charing-cross, have had the glasses ground and fitted for me, and make them at any angle which may be required. Messrs. Bland and Long, of Fleet street, also make them.

July 24, 1853.

---

NOTE.—Of three patients who have used the glasses, two have been greatly improved, and still go on favorably. In the third, where the power of the squinting eye was very much less than that of the opposite one, the strongest prism which could be worn without producing diplopia was ineffectual, and I had to recur to the old method of exercising the squinting eye while the other was covered. I do not use an ordinary shade or bandage as a covering, but have an India-rubber ring, which fits the orbit, covered on the outside with silk, and fastened by a ribbon. This allows free motion of the eye and eyelids, while the light is perfectly excluded.

